# Signaling events evoked by domain III of envelop glycoprotein of tick-borne encephalitis virus and West Nile virus in human brain microvascular endothelial cells

**DOI:** 10.1038/s41598-022-13043-1

**Published:** 2022-05-25

**Authors:** Katarína Bhide, Evelína Mochnáčová, Zuzana Tkáčová, Patrícia Petroušková, Amod Kulkarni, Mangesh Bhide

**Affiliations:** 1 Laboratory of Biomedical Microbiology and Immunology, University of Veterinary Medicine and Pharmacy, Komenského 73, 04181 Kosice, Slovak Republic; 2 Institute of Neuroimmunology of Slovak Academy of Sciences, Bratislava, Slovak Republic

**Keywords:** Molecular biology, Transcriptomics, Immunology, Infectious diseases, Microbiology, Virology, Virus-host interactions, West nile virus

## Abstract

Tick-borne encephalitis virus and West Nile virus can cross the blood–brain barrier via hematogenous route. The attachment of a virion to the cells of a neurovascular unit, which is mediated by domain III of glycoprotein E, initiates a series of events that may aid viral entry. Thus, we sought to uncover the post-attachment biological events elicited in brain microvascular endothelial cells by domain III. RNA sequencing of cells treated with DIII of TBEV and WNV showed significant alteration in the expression of 309 and 1076 genes, respectively. Pathway analysis revealed activation of the TAM receptor pathway. Several genes that regulate tight-junction integrity were also activated, including pro-inflammatory cytokines and chemokines, cell-adhesion molecules, claudins, and matrix metalloprotease (mainly ADAM17). Results also indicate activation of a pro-apoptotic pathway. TLR2 was upregulated in both cases, but MyD88 was not. In the case of TBEV DIII, a MyD88 independent pathway was activated. Furthermore, both cases showed dramatic dysregulation of IFN and IFN-induced genes. Results strongly suggest that the virus contact to the cell surface emanates a series of events namely viral attachment and diffusion, breakdown of tight junctions, induction of virus uptake, apoptosis, reorganization of the extracellular-matrix, and activation of the innate immune system.

## Introduction

Tick-borne encephalitis virus (TBEV) and West Nile virus (WNV) are the members of the family *Flaviviridae*, genus *Flavivirus.* Both infections can severely affect the central nervous system (CNS) in humans. The clinical manifestations of tick-borne encephalitis range from fever and meningitis to severe complications such as meningoencephalitis, encephalomyelitis, or paresis^1,2^. On the other hand, the majority of WNV infections result in a mild illness or remain asymptomatic. A small percentage of WNV-infected individuals experience severe neurological symptoms such as meningitis, acute flaccid paralysis, and encephalitis^3^. Both viruses enter into the CNS through the hematogenous route or axonal transport^4–6^. The hematogenous route is the most common, which involves crossing the blood–brain barrier (BBB)^6^. Encephalitis caused by WNV is characterized by disruption of the BBB, enhanced infiltration of immune cells into the CNS, microglial activation, inflammation, and eventual loss of neurons^7,8^, whereas, major hallmarks of TBEV associated encephalitis are neuroinflammation, disruption of the BBB^9–11^ and neuronal death^5,12,13^.

The BBB is composed of brain microvascular endothelial cells (BMECs) strongly adhered through tight junction proteins, in association to pericytes, astrocytes, and microglia. It is a highly selective semipermeable barrier that prevents non-selective passage of solutes and blood cells into the CNS. Pathogens, on the other hand, have evolved a variety of evasion strategies to cross the BBB. Some of the members of flavivirus can employ all three routes of translocation, i.e. the transcellular, paracellular and “Trojan horse” mechanisms (via infected leukocytes)^6^. TBEV and WNV, in particular, can cross the BBB via infected “Trojan horse” route and transcytosis^9^. It is also noteworthy that, the inflammatory mediators released during the systemic inflammation, in response to the flavivirus infection, may affect the permeability of the endothelial barrier allowing passage of viruses across BBB. Replication of WNV in the BMECs may induce downregulation of tight junction proteins, promoting the barrier disruption and virus entry^14^. After systemic infection, some flaviviruses like Japanese encephalitis virus (JEV), Zika virus (ZIKV), WNV and TBEV reaches the BBB and may cross the endothelial barrier as cell-free virus, without remarkable cytopathic effect^9,15–18^. Thus, evaluation of the interaction of cell-free virus with BMECs at transcriptomic level is of main interest here.

The cell entry of flaviviruses is initiated by the interaction of the E glycoprotein with cellular surface receptors. E glycoprotein has three structurally distinct domains (DI, DII and DIII)^19^, while the DIII is an immunoglobulin-like structure crucial in receptor binding and viral attachment to the cell surface^20^. It mediates interaction with glycoaminoglycans (GAGs, e.g. heparan sulfate)^21^, which leads to the concentration of virus at the cell surface. Several other attachment molecules like DC-SIGN/L-SIGN, laminin receptor, TIM receptors, TAM receptors, Integrin αvβ3 and NKp44 have been identified as receptors for viral attachment^22^ and the binding is proposed to be mediated through interaction of DIII with majority of the receptors^23^. An immunoglobulin-like fold of DIII is suggested to be involved in the cell attachment^24^, and mutations in this region showed alteration in the virulence^25–27^ underlying the importance of DIII in viral attachment and pathogenesis.

Since the cell-free TBEV and WNV can attach directly to the endothelial cells of the BBB and infect them, viral replication in BMECs can be one of the major routes of viral translocation across BBB. Cell-free WNV can cross the BBB either by transcytosis (i.e. without altering the BBB integrity), or by modulating tight junction integrity thereby allowing passage of virus into the CNS^28,29^. Previous study has shown that permeability of the BBB was increased at later stages of TBEV infection when the virus load in CNS was high, however breakdown of BBB integrity was not necessary for TBEV entry into the brain^9^. Similarly, it was proposed that WNV initially enters CNS without altering the BBB integrity and later virus replication in the brain initiates BBB disruption, allowing enhanced infiltration of immune cells and contribute to virus neuroinvasion via the ‘Trojan-horse’ route^30^.

Several studies have been conducted to examine cell signaling during flaviviral infections, in various cells lines and organs such as cerebrum and thalamus of horses^31^, brains of mice^32^, retinal epithelium^33^, human medulloblastoma cells derived from cerebellar neurons^34^, BMECs^14,17^ and single cell murine fibroblast^35^. However, to our knowledge, no study has revealed comprehensive response of the human BMEC (HBMECs) to protein E. The early stages of the cell infection by flavivirus involve receptor recognition, internalization and uncoating. These processes are seemingly simple, but their impact on the infected BMECs is not fully explored. To this background, we employed high-throughput RNA sequencing (RNA-seq) to elucidate complete picture of the signaling events triggered by the domain III of envelop glycoprotein of TBEV and WNV in HBMECs. Understanding the complete map of gene changes underlying the initial stages of HBMECs-virus crosstalk (receptor recognition and virus internalization) could help to develop potential therapeutic.

## Results and discussion

### Recombinant DIII (rDIII) of protein E of TBEV and WNV

Domain III of protein E of both viruses was overexpressed, purified, and used to challenge HBMECs. Amino acid sequences of DIII, purity of the recombinant protein judged by SDS-PAGE and molecular mass (~ 12 kDa) measured with MALDI-TOF/MS are presented in Supplementary Information Fig. S1. Both rDIII were in pure form, without non-specific proteins and degradation.

### RNA-seq analysis

RNA isolated from HBMECs were subjected to quality control before downstream library preparation and NGS analysis. A quality check performed on the capillary electrophoresis showed no signs of degradation (Supplementary Information Fig. S2). All cDNA libraries had optimal fragment size of 150–300 nt (Supplementary Information Fig. S3). Nine cDNA libraries were generated from 3 biological replicates as follows: non-induced HBMECs (NC1 to NC3), HBMECs induced with rDIII of TBEV (TBEV1 to TBEV3) or WNV (WNV1 to WNV3). In sequencing the average raw reads obtained for HBMECs exposed to rDIII of TBEV (rDIII-TBEV) were 1.3 × 10^7^, while for the cells induced with rDIII of WNV (rDIII-WNV) and mock control (negative control) it was 1.2 × 10^7^. In total, 11,398 genes for each treatment were mapped (Supplementary Datasets 1.1 and 1.2).

### Differentially expressed genes (DEGs) and validation

**Figure 1:**
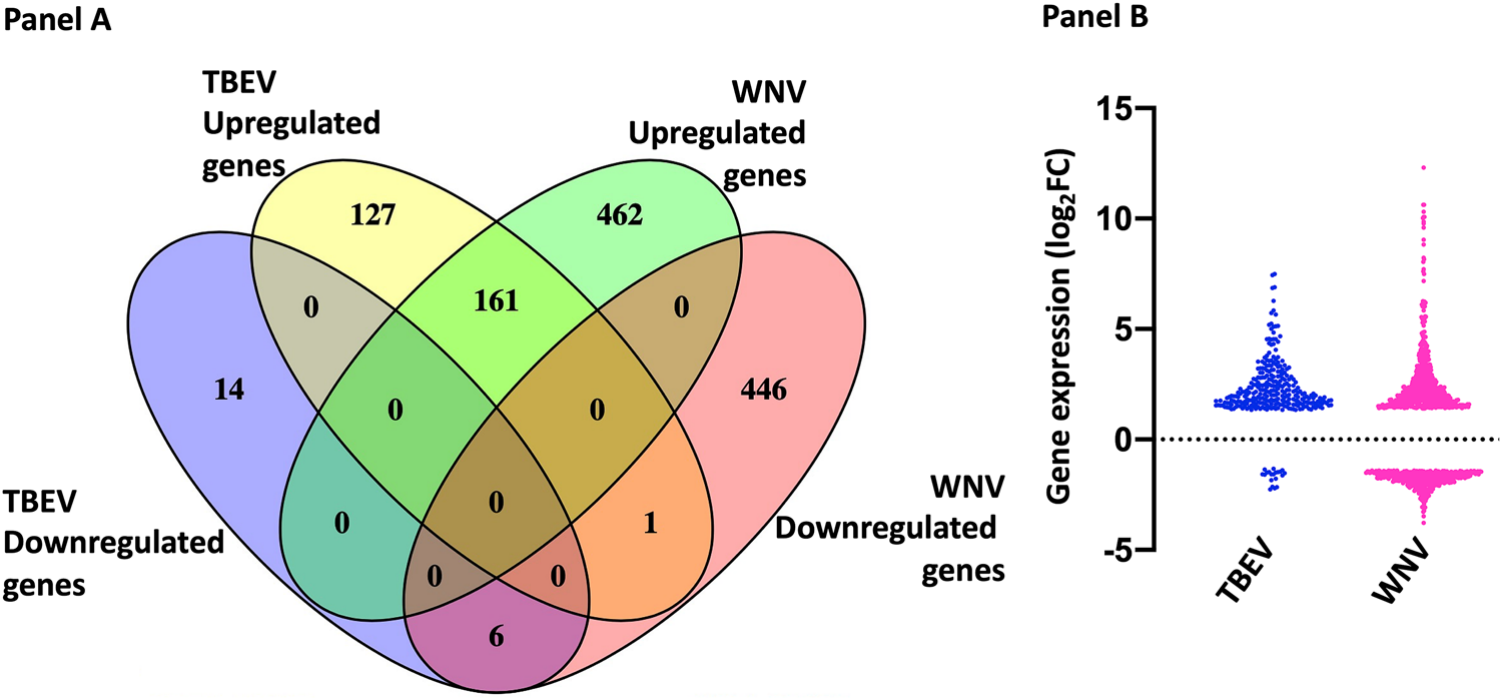
Differentially expressed genes (DEGs). (**A**) A Venn diagram showing number of DEGs evoked in HBMECs challenged with rDIII of TBEV or WNV. Yellow and blue ellipses represent up- and down-regulated DEGs in HBMECs incubated with rDIII-TBEV, respectively. Green and pink ellipses represent up- and down-regulated DEGs in HBMECs incubated with rDIII-WNV, respectively. Common DEGs are shown in the intersections. (**B**) A nested graph of the DEGs evoked in HBMECs. Each dot represents a gene. Log_2_fold change values observed in HBMECs induced with rDIII of TBEV and WNV are plotted in this graph.

Analysis of RNA-seq expression profiles performed with edgeR of Bioconductor revealed a total of 309 DEGs (289 up-regulated genes and 20 down-regulated) evoked in rDIII-TBEV treated endothelial cells, while 1076 DEGs (623 up-regulated genes and 453 down-regulated) were found in HBMECs challenged with rDIII-WNV (Fig. 1A; Supplementary Datasets 1.3 and 1.4). The maximum and minimum log_2_fold change values (logFC), resulting from averaging three independent biological replicates are presented in Fig. 1B. A total of 168 genes were evoked in a similar fashion in both treatments (161 up-regulated, 6 down-regulated), whereas one gene, RAB42 a member RAS oncogene family, was upregulated in HBMECs when treated with rDIII-TBEV and downregulated when challenged with rDIII-WNV (Fig. 1A; Supplementary Dataset 1.5). It is important to note that in transcriptome analysis, the level of gene expression and pattern of expression (between rDIII-WNV and rDIII-TBEV) may vary depending on the length of incubation of rDIII with HBMECs. Six hours of the incubation was performed in the present study to obtain reliable data of the transcripts encoding metalloproteases, proteins involved in reorganization of the extracellular matrix, proteins involved in the degradation of tight junction. In our preliminary experiments, challenging HBMECs for 1 or 3 h induced several transcripts related to the cell surface receptors and genes related to the innate immune system, however genes involved in alteration of BBB permeability were not fully evoked (data not shown). In case of longer incubation (12 and 18 h) expression of the gene related to cells senescence was highly evoked (data not shown).

**Figure 2:**
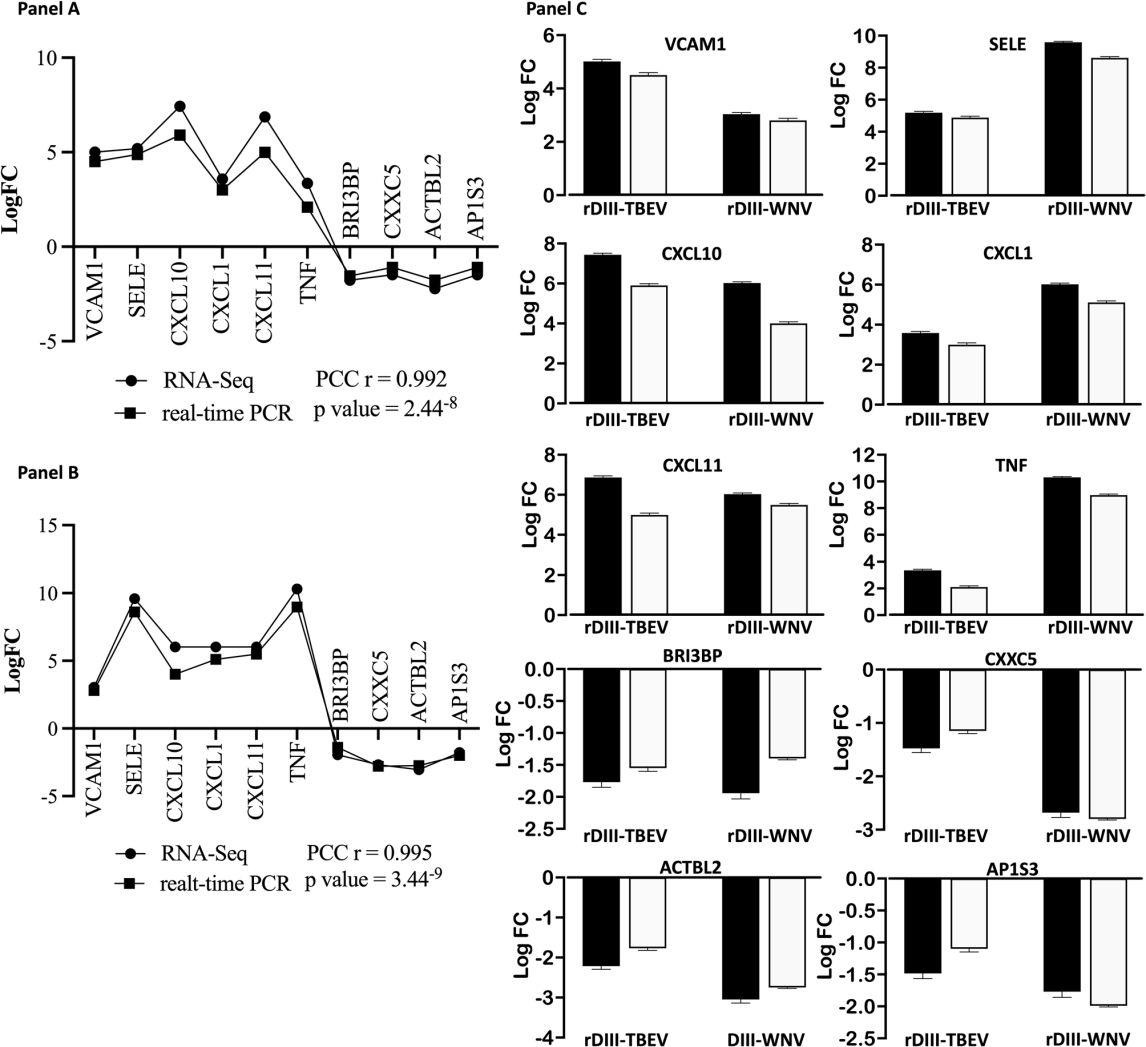
Validation of results obtained from RNA-seq with real-time PCR. (**A**) Gene expression (logFC) compared with linear regression (cell induced with rDIII-TBEV)*.* (**B**) Gene expression (logFC) compared with linear regression (cell induced with rDIII-WNV)*.* (**C**) Gene expression levels (logFC) obtained from RNA-seq and real-time PCR. Black bars—logFC obtained from RNA-seq, white bars—logFC calculated from real-time PCR.

To validate the results obtained from the RNA-seq analysis, a subset of 10 representative DEGs was analyzed with real-time PCR (Supplementary Information Table S1). Results were consistent to those obtained from RNA-seq with a high correlation, which was evaluated by the Pearson correlation coefficient (r = 0.992 for HBMECs exposed to rDIII-TBEV and r = 0.995 for HBMECs induced with rDIII-WNV, Fig. 2). This confirms reliability of data derived from RNA-seq analysis.

### Categorization of the DEGs according to the gene ontology (GO)-molecular function and GO-biological processes

Differentially expressed genes were categorized based on their involvement in different biological processes using a peer-reviewed Reactome server that uses enrichment analysis corrected for false discovery rate using the Benjamani–Hochberg method (https://reactome.org). A complete list of the GO-biological processes and pathways for each treatment found in the enrichment analysis is presented in Supplementary Datasets 1.6 and 1.7.

### Signaling events evoked in HBMECs in response to rDIII

The virus contact to host cell surface, emanates series of events namely viral attachment and diffusion^36^, breakdown of tight junctions^37^, and induced virus uptake and transcytosis^36^. Attachment can also evoke apoptosis^38^ and induce reorganization of the extracellular matrix^39–41^. Further, the attachment is often associated with disturbance in the cell physiology (increased level of stress proteins) and the activation of the innate immune system^42^. Below we have elaborated post-attachment biological events which we observed in the HBMECs in response to rDIII, and compared our results with available literature on the viral attachment, cell entry and cells response.

#### Viral attachment receptors

**Figure 3:**
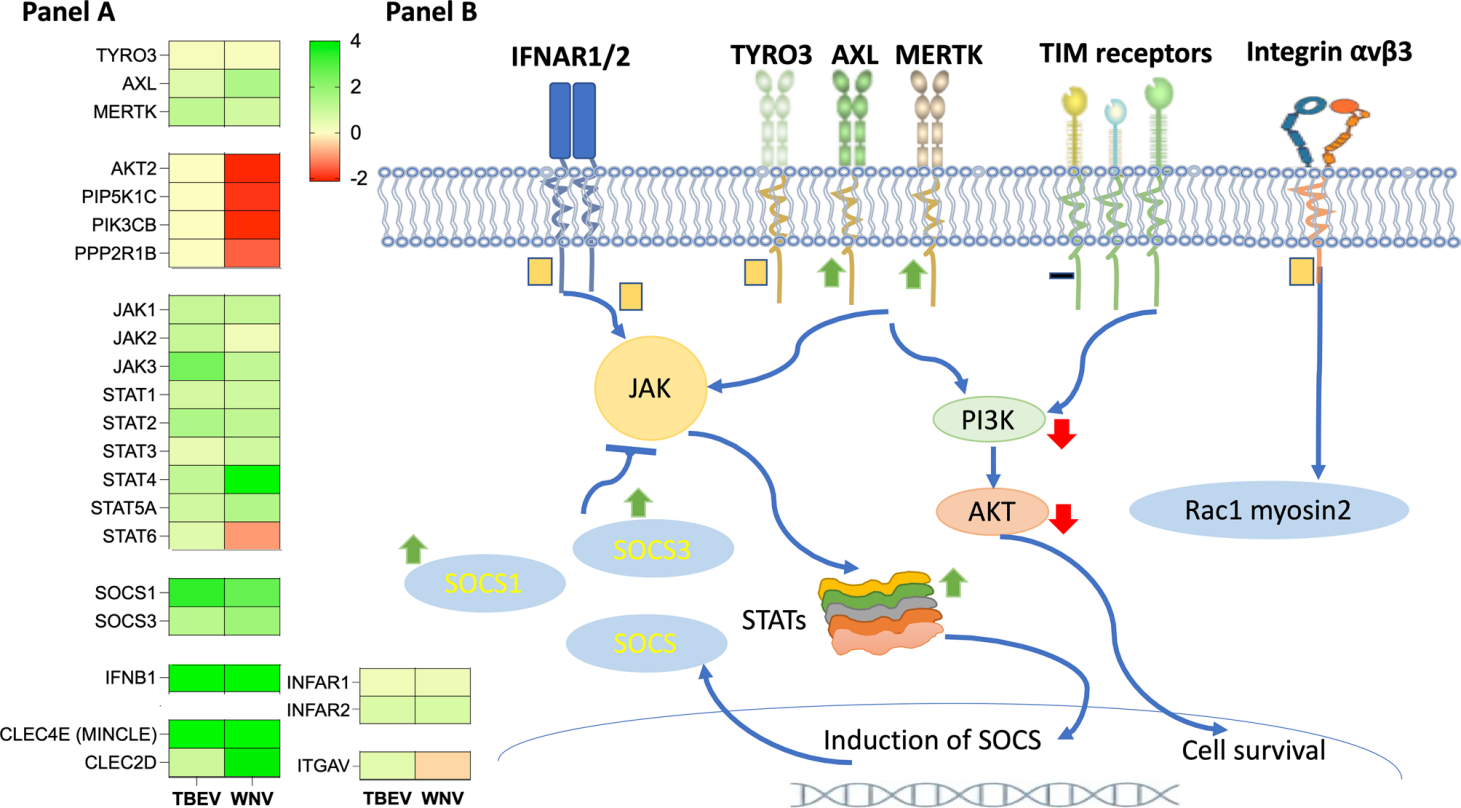
Viral attachment receptors. (**A**) Heat maps showing differentially expressed genes encoding viral attachment receptors and their downstream signaling molecules. The intensity of the color indicates the degree of expression level. Range of the logFC is presented in the scale. Gene expression was considered as significant only when the LogFC was beyond ± 1.2, average logCPM (count per million) was > 3, and *p*-value was < 0.01. An edgeR package was used for statistical analysis of differentially expressed gene. The unshrunk.logFC and false discovery rate (FDR) values for each genes are presented in Supplementary Dataset. (**B**) Attachment of flaviviruses to entry receptors evoke various pathways like endocytosis, changes in cell cytoskeleton via integrins/Rac-1-myosin2, cell survival signaling via PI3K-AKT (TIM and TAM receptors), suppression of immune response via JAK-STAT and activation of SOCS-1/3 (mediated through TAM receptors), which help for viral infectivity. Expression of IFNAR1, IFNAR2 and integrin αv did not altered in our study (yellow squares present no significant deregulation). None of the member of TIM family was detected (black dash indicates no detection of gene in our study). Expression of MERTK and AXL (TAM) were upregulated significantly by rDIII-TBEV and rDIII-WNV, respectively (green arrows indicate significant upregulation). Activation of TAM typically induces PI3K-AKT pathway. Note that, expression molecules in JAK-STAT signaling pathway (e.g. JAK3 and STAT4) were upregulated instead of genes in PI3K-AKT signaling (expression of AKT2, PIP5K1C, PIK3CB and PPP2R1B unchanged by rDIII-TBEV and downregulated by rDIII-WNV). Red arrows indicate downregulation of genes involved in given pathway. Activation of JAK-STAT promotes expression of SOCS1 and 3 (beneficial for the virus entry and replication). Both SOCS1 and SOCS3 genes were also upregulated by rDIII of TBEV or WNV.

Virion must attach to the cell surface to initiate the infection^24^. Two types of receptors have been described, the attachment factors and true receptors. Attachment factors bind to diverse moieties like phosphatidylserine and sugars on viral glycoprotein, while true receptors (entry factors) mediate interaction with viral proteins (protein E in case of flaviviruses) that leads to viral internalization^43^. In our study we looked at the expression of true receptors upon challenge with DIII of protein E. Amongst the true receptors supposed to be involved in flavivirus entry, the best characterized to date include TIM (T-cell immunoglobulin e.g. TIM-3, TIM-4), TAM (TYRO3, AXL and MERTK), integrin αvβ3 and C-type lectin receptors (e.g. CLEC4E and CLEC5A)^22,44,45^, while several additional molecules like IFNAR (type I interferon receptor) and HSPs (HSP70 and HSP90) are proposed as flaviviral attachment proteins. Hitherto, scantly literature is available on the deregulation in gene expression of cell receptors during the flaviviral infection^46,47^. In our study, expression of IFNAR1, IFNAR2 and integrin αv did not altered significantly in rDIII-TBEV and rDIII-WNV challenged cells (Fig. 3), while none of the member of TIM family was detected in RNA-seq. Taking into account that TIM-3 and TIM-4 are expressed only in antigen presenting cells^48^, absence of their transcripts in our study is not surprising. On the other hand, expression of the members of TAM family was evoked in endothelial cells after incubation with rDIII. MERTK was overexpressed in the cells incubated with rDIII-TBEV (logFC 1.2), while expression of AXL was increased 1.5 fold (logFC 1.5) in case of rDIII-WNV (Fig. 3). TAM signaling typically activates PI3K-AKT pathway leading to cell survival and proliferation^49^. However, it is proposed that in flavivirus infections TAM signaling leads to activation of JAK-STAT signaling followed by promotion of the expression of SOCS1 and 3 (suppressor of cytokine signaling) molecules^48,50^. Activation of SOCS molecules is beneficial for the virus entry and replication. As SOCS are known to act as a false substrate for phosphorylation or ubiquitinate transcription factors, their induction blocks further JAK-STAT activity and abrogates interferon-stimulated genes (ISG) to be translated^50–52^. High levels of type I interferons (mainly IFNβ) are necessary for triggering of above stated events^53^. In our study, most of the genes (AKT2, PIP5K1C, PIK3CB and PPP2R1B) related to PI3K-AKT were either downregulated or remain at the basal level of expression (Supplementary Datasets 1.3 and 1.4), whereas, the expression of the genes in JAK-STAT signaling pathway was induced significantly (Fig. 3). JAK3 was the most upregulated gene in rDIII-TBEV challenged cells (logFC 2.4), while the expression of STAT4 was substantially evoked (logFC 6.5) by rDIII-WNV. Both SOCS1 and SOCS3 genes were also upregulated in challenged cells (Fig. 3). In the background of these events high levels of gene expression of IFNB1, a type I interferon, was evident in both treatments (rDIII-TBEV—logFC 4.88, rDIII-WNV—logFC 10.64, Fig. 3). Series of orchestral gene regulation observed in our study and reported by others^51,52^, may indicates how the sheer attachment of virion (mediated through domain III of protein E) go beyond the receptor-protein E interaction and activates differential signaling pathways in infected cells.

#### Tight junction breakdown: deregulation of cell adhesion molecules and junctional proteins

Several reports indicate that WNV and TBEV cross the BBB^6,54,55^, however, whether they compromise the integrity of the BBB is still subject of several studies. Studies indicate various mechanisms of the disruption of tight junction e.g. downregulation of tight junction proteins (mainly claudin-5, occludin, and ZO-1) due to overproduction of pro-inflammatory cytokines and chemokines, such as IL-6, IFN-γ, CXCL10, CCL2 and CCL5^30^, degradation of tight junction proteins by induced matrix metalloproteinases^30,56^ and endocytosis of a subset of tight junction membrane proteins including claudin-1 and JAM-1, followed by lysosomal degradation of the proteins^57^. Along with altered expression of tight junction proteins, deregulation of the cell adhesion molecules (CAMs mainly, ICAM-1, VCAM-1 and E-selectin) also influences permeability of the BBB.

**Figure 4:**
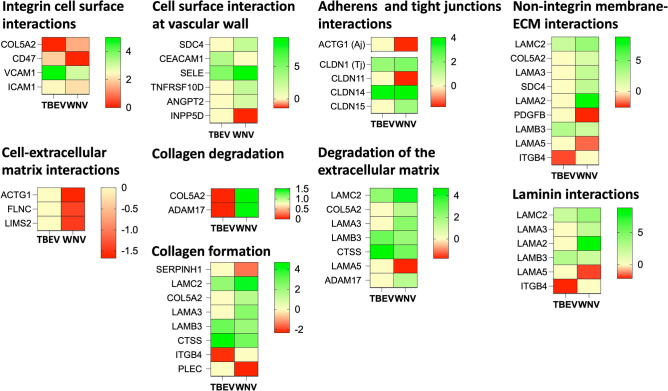
Deregulation of the genes involved in the reorganization of the extracellular matrix in the HBMECs induced with domain III of TBEV or WNV. Heat maps presenting deregulation of the gene expression involved in various GO biological processes that cause reorganization of extracellular matrix. The intensity of the color indicates the degree of expression level. Range of the logFC is presented in the scale. Gene expression was considered as significant only when the LogFC was beyond ± 1.2, average logCPM (count per million) was > 3, and *p*-value was < 0.01. An edgeR package was used for statistical analysis of differentially expressed gene. The unshrunk.logFC and false discovery rate (FDR) values for each genes are presented in Supplementary Dataset.

In our study, overexpression of pro-inflammatory cytokines and chemokines mentioned above was evident (IL-6—rDIII-TBEV—logFC 3.33, rDIII-WNV—logFC 4.66; CXCL10—rDIII-TBEV—logFC 7.43, rDIII-WNV—logFC 6.0; CCL2—rDIII-TBEV—logFC 5.14, rDIII-WNV—logFC 3.82; and CCL5—rDIII-TBEV—logFC 4.28, rDIII-WNV—logFC 4.05, Supplementary Datasets 1.3 and 1.4). We also observed significant upregulation of cell adhesion molecules (mainly ICAM-1, VCAM-1 and E-selectin, Supplementary Datasets 1.3 and 1.4, Fig. 4) in HBMECs induced with rDIII-TBEV as well as rDIII-WNV. The induction of cell adhesion molecules has an immediate impact on the migration of immunocompetent cells through the BBB. Particularly, in WNV infection permeability of the in vitro BBB model was increased following the transmigration of monocytes and lymphocytes^14^. Increased expression of ICAM-1, VCAM-1 or E-selectin was also observed in HBMECs infected by WNV^17^ and DENV^58^. Conversely, some authors have reported slight down-regulation ICAM-1, VCAM-1 and E-selectin in mice brains infected with WNV NY strain^59^ or TBEV^9,16^.

In our study, expression of tight junction proteins ZO-1 and occludin did not alter in induced cells compared to mock control (Supplementary Datasets 1.1 and 1.2). Whereas, expression of claudins (claudin-1, -11, -14, -15) was dramatically deregulated (Supplementary Datasets 1.3 and 1.4, Fig. 4) in both rDIII-TBEV as well as rDIII-WNV treated cells. In previous study, increased levels tight junction proteins were also observed in HBMECs infected with WNV^17^. In contrast, downregulation of tight and adherent junction proteins (claudin1, occludin, ZO-1 and JAM A), catenin beta or VE-cadherin were reported in WNV infection^30,60^. In case of TBEV infection of endothelial cells, expression of occludin and ZO-1 did not alter at any timepoint of investigation (from 12 to 40 h post infection)^16^.

Matrix metalloproteinases of MMP and ADAM family play important role in degradation of tight junction proteins, while several pathogens exploit metalloproteinases to cross the BBB^61^. In line with previous reports that propose a proteases mediated breakdown of tight junctions in flaviviral infection^17,30,37,54^, we expected significant increases in matrix metalloproteases such as MMP-3 and MMP-9. However, in our study none of the proteases of MMP family was significantly evoked. The only matrix metalloprotease significantly upregulated in our experiment was ADAM17 in rDIII-WNV challenged cells (logFC 1.46), while in rDIII-TBEV treated cells the change was non-significant (logFC 0.85) (Fig. 4).

#### Genes involved in the reorganization of the extracellular matrix (ECM)

The extracellular matrix is a highly dynamic structure that regulates a wide range of functions such as cell proliferation, migration, and differentiation^62^. It is shown that the Zika and dengue infection cause significant dysregulation of ECM composition^63–65^. However, data on the dysregulation of ECM components caused by other flaviviruses is still limited. Dengue virus infection mainly downregulates expression of PATJ (Protein associated to tight junctions) and CRTAP (Cartilage associated protein)^65^. In our study, CRTAP expression was downregulated (logFC − 0.64) in the case of rDIII-TBEV but not in the case of rDIII-WNV (logFC 0.27). PATJ expression remained unchanged in the HBMECs after both challenges.

When we analyzed a set of DEGs evoked by rDIII-TBEV or rDIII-WNV with pathway enrichment analysis, three pathways related to ECM reorganization were popped up, namely non-integrin membrane-ECM interactions (R-HSA-3000171), laminin interactions (R-HSA-3000157) and cell-ECM interactions (R-HSA-446353) (Fig. 4, Supplementary Datasets 1.6 and 1.7). The expression of three candidates from cell-ECM interaction, ACTG1 (actin gamma 1), FLNC (filamin C), and LIMS2 (LIM zinc finger domain containing 2), was significantly downregulated in cells incubated with rDIII-WNV; however, in rDIII-TBEV, the expression levels of all three candidates were similar to mock control (Fig. 4). In case of collagen degradation pathway both upregulated candidates (ADAM17 and COL5A2) in rDIII-WNV treated cells were negatively regulated in rDIII-TBEV challenged cells (Fig. 4). Other genes, including PDGFB, LAMA5, ITGB4, SERPINH1, and PLEC, also displayed a differential pattern of expression between treatments (Fig. 4). We have no explanation for the treatment-dependent differential gene expression pattern, but it is certainly worth investigating further in future studies.

In our study, dysregulation of genes encoding laminin subunits (LAMC2, LAMA3, LAMA2, LAMB3, and LAMA5) stands out (Fig. 4). LAMA2 was the most evoked gene (logFC 8.82) in the cells challenged with rDIII-WNV, with other laminin subunits: LAMC2 (logFC 4.00), LAMA3 (logFC 2.39) and LAMB3 (logFC 2.05). Interestingly, expression of LAMA2 and LAMA3 remained at basal level in rDIII-TBEV infected cells. In earlier study, upregulation of genes encoding laminin chains (LAMA2 LAMA3, LAMA4, LAMC3) was also observed in dengue infection^66^, while increased expression of LAMB2 was associated with dengue hemorrhagic fever^67^. Given that the protein E of TBEV can bind to laminin subunits (e.g. LAMB1) and use them to gain cell entry^68^, it is tempting to speculate that virus could use upregulation of laminin subunits to increase cell adhesion and entry.

#### Virus uptake: endocytosis and transcytosis

**Figure 5:**
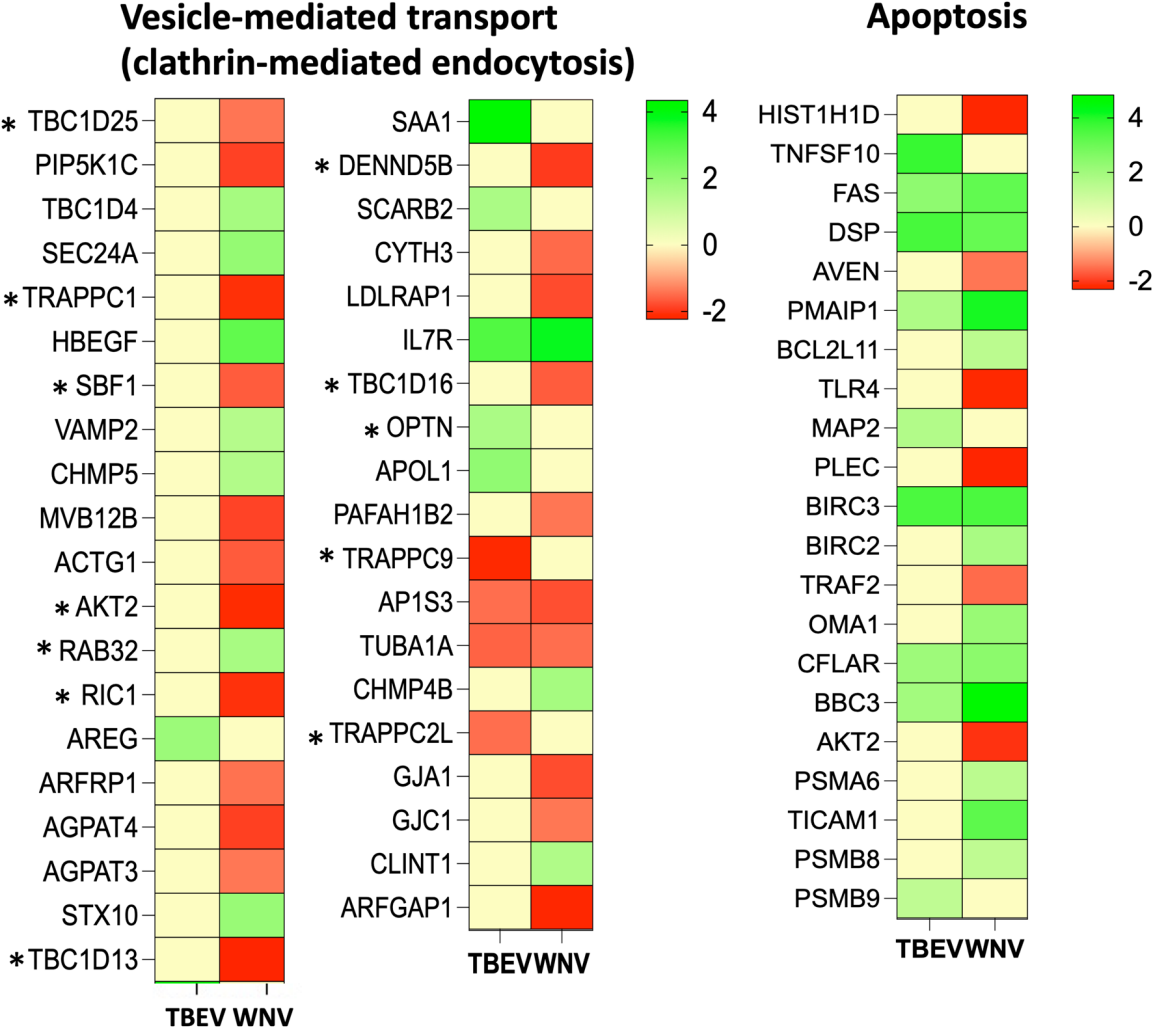
Differential expression of the genes in HBMECs challenged with rDIII-TBEV and rDIII-WNV. Heat maps presenting deregulation of gene expression involved the vesicle-mediated transport (mainly clathrin-mediated endocytosis and Rab regulation of trafficking). * Indicates genes involved in Rab regulation of trafficking. Attachment of rDIII on HBMECs also evoked expression of several genes related to apoptosis. The intensity of the color indicates the degree of expression level (logFC), presented in the scale. Gene expression was considered as significant only when the LogFC was beyond ± 1.2, average logCPM (count per million) was > 3, and *p*-value was < 0.01. An edgeR package was used for statistical analysis of differentially expressed gene. The unshrunk.logFC and false discovery rate (FDR) values for each genes are presented in Supplementary Dataset.

Most flaviviruses require endocytosis to traffic into a low pH compartment for fusion. In most of the cases, the pathway used by flaviviruses is clathrin-dependent uptake^43^. In our experimental data, among a set of genes related to clathrin mediated endocytosis, IL7R was the most significantly upregulated gene in both challenges (rDIII-TBEV—logFC 3.07, rDIII-WNV—logFC 3.73; Fig. 5). Apart from IL7R, AP1S3 and TUBA1A candidate, we saw no consistency in the expression pattern of the genes related to vesical mediated endocytosis evoked by rDIII-WNV and rDIII-TBEV. For example, ACTG1, PIP5K1C, LDLRAP1 and ARFGAP1 genes were significantly downregulated in rDIII-WNV challenged cells, while their expression after rDIII-TBEV challenge remained unaltered (Fig. 5, Supplementary Datasets 1.3 and 1.4).

Studies have reported interaction of WNV E glycoprotein with α_v_β_3_ integrin, which activates the PI3 kinase (PI3K) that in turn induces the synthesis of phosphatidylinositol 3,4-bisphosphate and phosphatidylinositol (3,4,5)-trisphosphate. These phosphoinositides are subsequently responsible for the activation of protein kinase C (PKC) and small GTPases such Rabs and Rho. GTPases then promote clathrin-dependent uptake of viral particles^69^. Recent studies have indicated importance of Rab protein family during internalization of flavivruses^43,70^, and the majority of viral particles enters Rab5-positive early endosomes^71^. Interestingly, in the present study Rab32 was upregulated in rDIII-WNV treated cells (logFC 1.66), however other genes involved in Rab regulation of trafficking were either downregulated or their expression remained at basal level in both rDIII-WNV or rDIII-TBEV treated cells (Fig. 5).

#### Apoptosis

Several viral proteins have been shown to regulate apoptosis^72^. While some proteins (like core protein) of flaviviruses induce pro-survival signals^73^, other, E and M in particular were reported to induce pro-apoptotic activity^74,75^. Glycoprotein E of the dengue, Zika and Japanese encephalitis viruses have been shown to induce programmed cell death^75–77^. Thus, we speculated that we might see deregulation of several genes related to apoptosis in our study. It is interesting to note that, 18 pro-apoptosis genes were evoked by rDIII-WNV, while rDIII-TBEV could deregulate only 9 genes (Fig. 5). While none of the gene was downregulated in rDIII-TBEV treated cells (Fig. 5), six genes were observed significantly downregulated in case of rDIII-WNV. Among those downregulated genes, three genes tightly regulate apoptosis namely: AKT2 (logFC − 2.11, regulates cell survival via the phosphorylation of MAP3K5), AVEN (logFC − 1.46, apoptosis and caspase activation inhibitor), and TRAF2 (logFC − 1.55, interacts with the inhibitor-of-apoptosis proteins, and functions as a mediator of the anti-apoptotic signals from TNF receptors).

Among several pro-apoptotic deregulated genes, PMAIP1, BBC3, FAS, DSP and CFLAR were the most significantly upregulated in both rDIII-TBEV and rDIII-WNV treated cells (Fig. 5). PMAIP1 (rDIII-TBEV—logFC 1.75; rDIII-WNV—logFC 4.09) promotes activation of caspases and apoptosis, BBC3 (rDIII-TBEV—logFC 1.96; rDIII-WNV—logFC 4.83) cooperates with activator proteins to induce mitochondrial outer membrane permeabilization and apoptosis, FAS (rDIII-TBEV—logFC 2.34; rDIII-WNV—logFC 3.14) involve in the formation of a death-inducing signaling complex that plays central role in the programmed cell death, DSP (rDIII-TBEV—logFC 3.55; rDIII-WNV—logFC 3.07) causes apoptotic cleavage of cellular proteins, and CFLAR (rDIII-TBEV—logFC 2.04; rDIII-WNV—logFC 2.41) induces the apoptosis (Supplementary Datasets 1.3 and 1.4). Altogether, results strongly indicate that attachment of the glycoprotein E on the endothelial cells, mediated through the DIII, induces pro-apoptotic pathway.

#### Activation of the innate immune system

**Figure 6:**
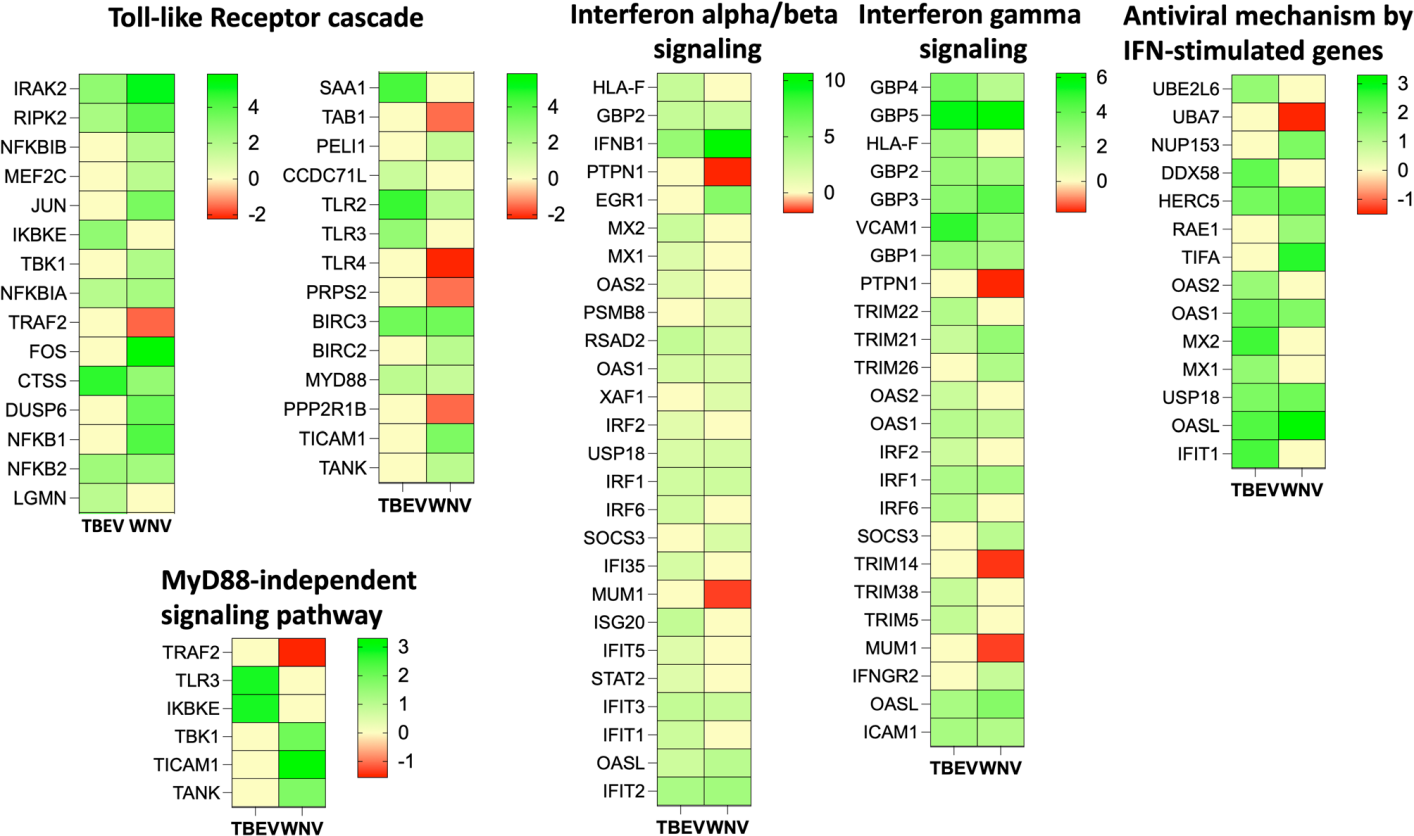
Deregulation of the genes in HBMECs challenged with rDIII of TBEV and WNV. Heat maps presenting differential expression of the genes involved in the TLR and interferon signaling pathways. Viral attachment protein evokes immediate cell response in non-immune cells, mediated through patter recognition receptors. Note the high upregulation of IFNB1 in interferon alfa/beta signaling in response to rDIII of WNV, and deregulation of GBP3 and GBP5 genes in interferon gamma signaling in response to rDIII of both viruses. The intensity of the color indicates the degree of expression level (logFC), presented in the scale. Gene expression was considered as significant only when the LogFC was beyond ± 1.2, average logCPM (count per million) was > 3, and *p*-value was < 0.01. An edgeR package was used for statistical analysis of differentially expressed gene. The unshrunk.logFC and false discovery rate (FDR) values for each genes are presented in Supplementary Dataset.

One of the most immediate cell reactions to a viral infection is the immune response. An initial signaling is mounted immediately after binding of viral ligand to receptor by setting off an innate immune response to protect the cell. Although the HBMECs are not primary immune cells, several signaling events related to the innate immune response are evoked in response to the attachment of pathogen or its ligands on cell surface^78–80^. In the present study we found deregulation of several genes involved in immediate cell response mediated through pattern recognition receptors (PRRs) and downstream interferon signaling (Fig. 6).

##### Toll-like receptors

To date, four major classes of viral PAMPs (Pathogen Associated Molecular Patterns) have been identified to activate PRRs, that are the CpG DNA, ssRNA, dsRNA and envelope glycoproteins^81^. The glycoproteins play critical roles in the induction of innate immune response as they are sensed by the PRRs immediately after viral attachment^81^. In the present study, the rDIII of glycoprotein E of both viruses was recognized by TLR2 resulting into its induction (rDIII-TBEV—logFC 4.54 and rDIII-WNV—logFC 1.77). The primary adapter molecule MyD88 was also upregulated (rDIII-TBEV—logFC 1.74 and rDIII-WNV—logFC 1.48) resulting in the activation of both subunits of NF-κB (NF-κB1 and 2) and inflammatory cytokines viz. TNF, IL-1β and IL-6 (Supplementary Dataset 1.5, Fig. 6). In previous studies, activation of the TLR2 by soluble form of envelope glycoprotein of cytomegalovirus^82–84^, hemagglutinin of measles virus^85^, and envelope glycoprotein of herpes simplex virus-1^86^, leading to the induction of NF-κB and inflammatory cytokine secretion has been documented. It is important to note that, in our study TLR4 was downregulated in HBMECs treated with rDIII-WNV (logFC − 2.23) and its expression remained unchanged in case of rDIII-TBEV (Fig. 6). This indicates that the elicitation of TLRs was not due to the possible contamination of the endotoxins in purified rDIII.

A pathway enrichment analysis of the dataset obtained in our study also indicated activation of MyD88-independent signaling pathway. The key adapter molecule in this pathway that transduces signals, a TICAM1 (TIR-domain-containing adapter-inducing interferon-beta), was upregulated by rDIII-WNV (logFC 3.23), but not by rDIII-TBEV (Fig. 6). Induction of the TICAM1 is well known to stimulation of distinct pathways leading to the production of type I interferons and proinflammatory cytokines^87^. Similarly, it is known that MyD88-independent pathway is activated by sensing of PAMP by TLR3. In our study, the TLR3 was upregulated in rDIII-TBEV challenge (logFC 2.71), while its expression remained unchanged (logFC 0.07) in rDIII-WNV treated cells (Fig. 6).

TLR3 has been identified in variety of intracellular structures such as endoplasmic reticulum and endosomes, and it is known to recognize dsRNA, poly(I:C) and damaged-associated molecular patterns^88,89^. The upregulation of TLR3 by rDIII of glycoprotein E was unexpected in our study because envelop proteins have not previously been reported as TLR3 ligands. In the literature we found reports on upregulation of TLR3 in the early stages of flaviviral infections such as dengue virus^90^, WNV^91^ and Zika virus^92^, which augmented production of IFN-α and IFN-β^91,93^. However, in contrast to our experimental setup, virions were used to challenge the cells in previous reports^90–92^, so we believe that induction of TLR3 by protein E is worth further investigation.

##### IFN and IFN-stimulated genes

It was also proposed that, some viral glycoproteins are sensed by the cells immediately after viral attachment on the cell surface to produce IFN rapidly and induce expression of IFN-stimulated genes, thereby preparing the cell to counter a possible viral infection^94–97^. Relevant to these findings, we found in our study that rDIII of E glycoproteins of both flaviviruses were sensed by HBMECs and the expression of several genes in IFN α/β signaling pathway and IFNγ signaling pathway were induced (Fig. 6). Among all IFN related genes expression of IFNB1 (rDIII-TBEV—logFC 4.88, rDIII-WNV—logFC 10.6) and IFNL1 (rDIII-TBEV—logFC 2.42, rDIII-WNV—logFC 9.59) was profoundly increased. Total 20 genes in interferon alpha/beta signaling pathway (Type I IFN) were evoked by rDIII of TBEV; while in case of WNV 15 genes related to type I IFN were evoked (Fig. 6). Among these candidates, the interferon regulatory factor 1 (IRF1) was upregulated in both cases (rDIII-TBEV—logFC 2.22, rDIII-WNV—logFC 2.38). In previous report, it was shown that IRF1 activated transcription of genes involved in the response against viral infections^98^. In our study, transcripts of several IFN-stimulated proteins, mainly interferon induced protein with tetratricopeptide repeats (IFITs), were upregulated in challenged HBMECs (Fig. 6). IFIT1, IFIT2, IFIT3 and IFIT5, evoked by the rDIII (Fig. 6), are the known antiviral defense molecules in the cells, that take part in the inhibition of the viral replication and translation initiation^98^. It is widely believed that viral nucleic acid, which accumulate during the replication, triggers IFN production. However, a microarray analysis of dsRNA-treated cells showed lack all type I IFN genes^99^. On the other hand, viral attachment proteins mainly envelop glycoprotein^96^, non-replicating mutant^100^ and UV-inactivated virus^101^ were able to evoke the regulation of a substantial subset of the same genes that are dysregulated during a productive infection. These reports and our results on dysregulation of IFN and IFN-induced genes by rDIII of glycoprotein E highlights important aspects of immediate cell response to the viral attachment.

#### Deregulation of non-coding RNAs

To our knowledge, there is scanty literature on the expression of non-coding RNAs in viral infections, and almost no study has reported deregulation of these RNA species in the early stages of flavivirus infection. Few studies have shown that viral infection can alter expression of antisense RNA, mitochondrially encoded RNAs (mtRNAs), long noncoding RNAs (lncRNAs), long intergenic noncoding RNAs (lincRNAs), small nucleolar RNAs, etc.^102,103^. In our study several species of noncoding RNAs were evoked (Supplementary Datasets 1.3 and 1.4), wherein the deregulation of lncRNAs was predominant. Seven lincRNAs were upregulated in endothelial cells challenged with rDIII-TBEV, whereas in case of rDIII-WNV nearly 34 lincRNAs were deregulated. In both cases lncRNA BISPR (rDIII-WNV—logFC 2.11 and rDIII-TBEV—logFC 1.57) and the SCAMP1-AS1 (rDIII-WNV—logFC 2.76 and rDIII-TBEV—logFC 2.311) were significantly upregulated. In earlier study, increased expression of BISPR was also reported in influenza A virus (IAV) and hepatitis C virus (HCV) infected cells^104^ and it was hypothesized that BISPR might be involved in regulating viral infection by increasing the expression of antiviral protein BST2^105^. On the other hand, lncRNA PSMB8-AS1 was found necessary to enhance IAV replication and growth and its upregulation in IAV infected cells was reported earlier^106^. In our study, expression PSMB8-AS1 was also increased in rDIII-WNV treated HBMECs (logFC 2.15).

Among few downregulated noncoding genes, lncRNAs NEAT1, linc00294 and linc00205 were the significantly deregulated genes in rDIII-WNV, while none of the lncRNA was downregulated in rDIII-TBEV treated cells (Supplementary Datasets 1.3 and 1.4). To our knowledge no data is available on the significance of linc00294 and linc00205 in viral infection, however silencing of NEAT1 was linked to the enhanced Hantaan orthohantavirus (HTNV) replication^107^. In contrast to the downregulation observed in our study in rDIII-WNV infected cells (logFC − 1.8), other studies found upregulation of the expression of NEAT1 in HTNV, herpes simplex virus, HIV, IAV, rabies virus and Japanese encephalitis virus infected cells (reviewed in^108^).

## Conclusion

Several viruses induce cell signaling events immediately after attachment. Because the DIII domain of protein E is the only domain that mediates virion-cell attachment in the majority of flaviviruses, the post-attachment signaling events in HBMECs induced by domain III were thoroughly investigated in this study. Several genes involved in tight junction destabilization, virus uptake and transcytosis, apoptosis, extracellular matrix reorganization, stress, and the innate immune system were significantly evoked. Interestingly, DIII induced regulation of the members of TAM family, which activated JAK-STAT signaling and SOCS molecules rather than the PI3K-AKT pathway. Overexpression of pro-inflammatory cytokines and chemokines, matrix metalloprotease (primarily ADAM17 in the case of rDIII-WNV), deregulation of laminin subunits, and genes associated with collagen degradation suggest that extracellular matrix arrangement and tight-junction integrity may be altered following viral attachment. It is worth noting that DIII of WNV induces the expression of pro-apoptotic genes. The rDIII of WNV also significantly elicited genes involved in vesicle-mediated transport, but not rDIII of TBEV. Elicitation of an innate immune response, primarily via TLR and interferon pathways, was evident. We believe that the systemic dissection of signaling events revealed in this study will aid researchers in understanding the underlying molecular processes that occur immediately after attachment of TBEV and WNV to the building block of BBB—the HBMECs.

## Materials and methods

### Culture of HBMECs

Details of the cell culture are in presented in the Supplementary Information Method S1.

### Synthesis of recombinant rDIII

Goshawk strain of WNV and Hypr strain of TBEV were used in this study. Goshawk strain used in this study was isolated in Hungary in 2004 and is related to central African lineage 2 viruses. It is pathogenic and causes encephalitis^109^. Hyper strain of TBEV is isolated from neural tissues and deposited in European Virus Archive (EVA—https://www.european-virus-archive.com/virus/tbev-hypr). RNA isolated from these strains was reverse transcribed and cDNA was used for amplification of DIII of protein E. Sense and antisense primer sequences, overhangs of restriction sites used for downstream cloning and amplicon length are shown in Supplementary Information Table S2. Amplified fragments were gel purified, digested and ligated into pQE-30-mCherry plasmid. Details of the ligation, the vector, selection of clones, overexpression of the rDIII, purification and quality check with SDS-PAGE and MALDI are described in details in our previous publication^110^ and in Supplementary Information Method S2 to S4. Protein concentration was measured by Bradford method and aliquots of purified proteins were stored at − 80 °C until use.

### Challenge of HBMECs

HBMECs were cultured in 6 well plates till 80% confluency. Cells were washed with EBM-2 medium (without serum) and incubated either with rDIII of TBEV or rDIII of WNV (1 nmol/well) resuspended in EBM-2 medium (containing only serum, and l-glutamine) or with medium only (mock control, non-induced control) for 6 h at 37 °C under 5% CO_2_ atmosphere. Culture media was removed after incubation and HBMECs were subjected to RNA isolation.

### RNA isolation from HBMECs, library preparation and RNA sequencing

mRNA from HBMECs was isolated using RNeasy Mini Kit (Qiagen, Germany) and RNA were treated with DNase I (Qiagen) as per manufacturer’s instructions. Integrity of RNA was monitored by capillary electrophoresis (Fragment analyzer, Advanced Analytical Technologies, Inc., USA). RNA concentrations were quantified by nanodrop (Thermo Fisher Scientific). Samples were stored at − 80 °C until use.

Libraries for RNA sequencing were prepared exactly as described in our previous publication^80^ using QuantSeq 3′ mRNA-Seq Library Prep Kit (Lexogen, Austria). Details of library synthesis are presented in Supplementary Information Method S5. Libraries were sequenced on Illumina NextSeq, single-end 75 bp, to a minimal depth 8 million reads per sample.

### Bioinformatic analysis

STAR aligner was used to process Fastq files, aligned to reference genome (*Homo sapiens* GRCh38) and generate gene counts. Differential gene expression analysis was carried out by R package edgeR. Data segregation to generate the final relation of differentially expressed genes (DEGs) between the challenged HBMECs was performed using Excel (MS office). DEGs were selected by applying following parameters: average logCPM (count per million) > 3, log_2_FC (fold change) beyond ± 1.2 and *p*-value < 0.01.

Raw RNA-seq data and processed data showing DEGs were deposited to EBI Arrayexpress repository (https://www.ebi.ac.uk/arrayexpress/) under accession number E-MTAB-8052.

Venn diagram was created to display the relation between the transcriptomes (http://bioinfogp.cnb.csic.es/tools/venny/). Differentially expressed genes were categorized based on their involvement in different biological processes using a peer-reviewed Reactome server (https://reactome.org). Heat mapping function of Prism 9 (Graphpad, USA) was used to compare expression level of the DEGs involved in GO biological processes.

### Validation of differentially expressed genes by real-time PCR

One microgram of RNA and 100 pmol of random hexamer were mixed and heated at 65 °C for 5 min. Subsequently, 4 μL 5 × reaction buffer, 2 μL dNTP (10 mM), 1 μL RevertAid reverse transcriptase (200 U) and 0.5 μL RiboLock RNase inhibitor (20 U) (all components from Thermo Fisher Scientific, USA) were added. The reaction mixture was incubated 10 min at 25 °C followed by 1 h at 42 °C and 70 °C for 10 min.

For validation 10 DEGs were selected which showed significant up or down regulation in RNAseq. Primers for real-time PCR (Supplementary Information Table S1) were designed using Geneious Pro software (Biomatters, USA). Reaction mix for PCR was composed of 6 ng of cDNA, 1 × qPCR GreenMaster with highROX (Jena Bioscience, Germany), gene specific primers (10 pmol each) and RNase free water up to total volume 20 μL. Amplification cycle was as follows: 95 °C—10 min, 40 × 95 °C—15 s, 50–60 °C—30 s (annealing temperature varied according to the primers used), 72 °C for 30 s (signal capture), melting curve 60–95 °C—0.3% temperature increment/s (StepOnePlus, Thermo Fisher Scientific, USA). The gene expression (ΔΔCt) was normalized to β-2-microglobulin (house-keeping gene) as described before^80^. ΔΔCt values were converted to logFC (http://www.endmemo.com/algebra/log2.php). Correlation of expression values for DEGs obtained from RNA-seq and real-time PCR was determined by calculating the Pearson correlation coefficient (r^2^). Correlation plots and Pearson correlation were performed by Prism 9 software (Graphpad).

### Level of biocontainment used in the study

Experiments with HBMECs (cell culture and rDIII challenge) and *E. coli* (overexpression of rDIII) were performed in BSL-2 laboratories. All other experiments were performed in BSL-1 facility. *E. coli* producing rDIII were handled as per the regulations set for the work with genetically modified organisms by the ministry of environment of Slovak Republic (GMO class I risk factor).

## Supplementary Information

Supplementary Information 1.

Supplementary Information 2.

## Data Availability

The datasets generated and/or analyzed during the current study are available in the EBI Arrayexpress repository (https://www.ebi.ac.uk/arrayexpress/) under accession number E-MTAB-8052.
