# Supplementary material for: Signaling events evoked by domain III of envelop glycoprotein of tick-borne encephalitis virus and West Nile virus in human brain microvascular endothelial cells
Source: Sci Rep. 2022 May 25;12:8863. doi: 10.1038/s41598-022-13043-1 (PMC9133079; doi:10.1038/s41598-022-13043-1)
Supplement: Supplementary file 1 — Supplementary Information 1. [file 41598_2022_13043_MOESM1_ESM.docx]

**Signaling events evoked by domain III of envelop glycoprotein of tick-borne encephalitis virus and West Nile virus in human brain microvascular endothelial cells**

**Katarína Bhide^1^, Evelína Mochnáčová^1^, Zuzana Tkáčová^1^, Patrícia Petroušková^1^, Amod Kulkarni^1,2^, and Mangesh Bhide^1,2^**

**SUPPLEMENTARY FIGURES**

**Supplementary information Fig. S1. Production of recombinant DIII.**

**Panel A**. Amino acid residues encompassed in the recombinant form of rDIII of WNV and TBEV. **Panel B**. PCR products of amplified fragment encoding rDIII, resolved on the agarose gel. **Panel C**. Purified rDIII proteins separated on SDS-PAGE. **Panel D**. Molecular masses of the rDIII of WNV and TBEV confirmed with MALDI-TOF/MS.

**Supplementary information Fig. S2. Integrity of RNA.** RNA isolated from non-induced HBMECs (negative control, NC in triplicate) and HBMECs incubated either with rDIII of TBEV (TBEV1 to 3) or rDIII of WNV (WNV1 to 3).

**Supplementary information Fig. S3.** Capillary electrophoresis for the quality control of cDNA libraries prepared for RNA-seq. Nine cDNA libraries for sequencing were prepared with QuantSeq 3’ mRNA kit corresponding to non-induced HBMECs (negative control, NC1 to 3) and HBMECs incubated either with rDIII of TBEV (TBEV 1 to 3) or WNV (WNV 1 to 3). Ideal fragment size should be between 150-300 bp.

**Supplementary Fig. S4. Original photograph of the agarose gel used to make panel B of Supplementary Fig. S1**

***** - clones carrying undigested plasmid or without insert. **+** - DNA from undigested plasmid was used as template (positive control for PCR reaction). Lanes 4 and 6 were used in panel B of supplementary Fig. S1.

**Supplementary Fig. S5. Original photograph of the polyacrylamide gel (SDS-PAGE) used to make panel C of Supplementary Fig. S1.** Lane number 6 and 11 were used in panel C of supplementary Fig. S1. MW – molecular weight marker, 1 – whole cell lysate of *E.coli* fractionated on the gel after overexpression; 2 and 3 – flowthrough and washing from nickel affinity chromatography, respectively, 4 and 5 – elutions of rDIII of TBEV from nickel affinity chromatography, 6 – rDIII after gel filtration, 7, 8, 9 – elutions of rDIII of WNV from nickel affinity chromatography, 10, 11 – fractions after gel filtration of rDIII of WNV.

**Supplementary Fig. S6. Original spectrum (MALDI-TOF-MS) used to make panel D of Supplementary Fig. S1.**

Upper spectrum – rDIII of WNV, lower spectrum – rDIII of TBEV.

**Supplementary Fig. S7. Original photograph used to make Supplementary Fig. S2.**

Integrity of RNA. RNA isolated from non-induced HBMECs (negative control, NC in triplicate). Marker on the both side of picture shows molecular mass from 15 bp to 6000 bp

**Supplementary Fig. S8. Original photograph used to make Supplementary Fig. S2.**

Integrity of RNA. RNA isolated from HBMECs incubated either with rDIII of TBEV (TBEV1 to 3) or rDIII of WNV (WNV1 to 3). Marker on the both side of picture shows molecular mass from 15 bp to 6000 bp

**Supplementary Fig. S9. Original photograph used to make Supplementary Fig. S3.** Capillary electrophoresis for the quality control of cDNA libraries prepared for RNA-seq. cDNA libraries for sequencing were prepared with QuantSeq 3’ mRNA kit. HBMECs incubated with rDIII of TBEV (TBEV 1 to 3). Marker on the left side of picture shows molecular mass from 1 bp to 6000 bp. Ideal fragment size should be between 150-300 bp.

**Supplementary Fig. S10. Original photograph used to make Supplementary Fig. S3.** Capillary electrophoresis for the quality control of cDNA libraries prepared for RNA-seq. cDNA libraries for sequencing were prepared with QuantSeq 3’ mRNA kit corresponding to non-induced HBMECs (negative control, NC1 to 3) and HBMECs incubated with rDIII WNV (WNV 1 to 3). Marker on the left side of picture shows molecular mass from 1 bp to 6000 bp. Ideal fragment size should be between 150-300 bp.

| **Supplementary information Table S1**. **Primers used in real-time PCR** | | | | |
| --- | --- | --- | --- | --- |
| **Gene** | **Primer** | **Sequence (5´- 3´)** | **Annealing temperature** | **Amplicon length**  **(bp)** |
| β-microglobulin (house-keeping gene) | *b2m*- sense | GCTCGCGCTACTCTCTCTTT | 134 | 55 |
|  | *b2m*- antisense | CGGATGGATGAAACCCAGACA |  |  |
| Vascular cell adhesion molecule 1 | *VCAM1*- sense | CCCTGAGCCCTGTGAGTTTT | 138 | 60 |
|  | *VCAM1*- antisense | GGCCACCACTCATCTCGATT |  |  |
| Selectin E | *SELE-* sense | TGTGGAATGATGAGAGGTGCA | 138 | 60 |
|  | *SELE-* antisense | TGAAGCCAGGGTCACACTTG |  |  |
| CXCL10 (C-X-C motif chemokine ligand 10) | CXCL10- sense | GCCATTCTGATTTGCTGCCTT | 179 | 55 |
|  | CXCL10- antisense | GCAATGATCTCAACACGTGGAC |  |  |
| CXCL1 (C-X-C motif chemokine ligand 1) | *CXCL1-sense* | AGTGTGAACGTGAAGTCCCC | 101 | 60 |
|  | *CXCL1-antisense* | ATGGGGGATGCAGGATTGAG |  |  |
| CXCL11 (C-X-C motif chemokine ligand 11) | *CXCL11-sense* | CCCTGGGGTAAAAGCAGTGA | 149 | 60 |
|  | *CXCL11-antisense* | GCCTTGCTTGCTTCGATTTG |  |  |
| TNF (Tumor necrosis factor) | *TNF-sense* | CCTGTAGCCCATGTTGTAGCA | 154 | 55 |
|  | *TNF-antisense* | GGACCTGGGAGTAGATGAGGT |  |  |
| BRI3 binding protein | *BRI3BP-sense* | GAGATTTGTGCTGGGAGTGGA | 113 | 60 |
|  | *BRI3BP-antisense* | CTGGGCTGAAATACTGGGACA |  |  |
| CXXC finger protein 5 | *CXXC5-sense* | TGCAGCAGTTGTAGGAATCGA | 105 | 60 |
|  | *CXXC5-antisense* | AAGCATCACCTTCTCCAGAGC |  |  |
| Actin beta like 2 | *ACTBL2-sense* | GTTCGAGGCTTTCAACACACC | 106 | 60 |
|  | *ACTBL2-antisense* | CCCATCCCCAGAATCCATCAC |  |  |
| Adaptor related protein complex 1 sigma 3 subunit | *AP1S3-sense* | TATTCTCTCCCGTGGTCACAG | 141 | 60 |
|  | *AP1S3-antisense* | TGCACAATCTCTAGCGTCAAGA |  |  |

| **Supplementary information Table S2**. **Primers used to produce recombinant forms of rDIII** | | | | | | |
| --- | --- | --- | --- | --- | --- | --- |
| **Protein** | | **Sequence used to design primers –**  **Genbank accession number** | **Primer** | **Sequence (5´- 3´)** | **Amplicon length**  **(bp)** | |
| rDIII-TBEV | | AM600965 | *rDIII-TBEV-F* | CCGATATCGAAAGGTCTTACGTACACAATGTGTG | 342 | |
|  |  |  | *rDIII-TBEV-R* | GTGGTCGACTTCTATGCCCTTCTTGGTCTT |  |  |
| rDIII-WNV | | NC_009942 | *rDIII-WNV-F* | TTTGGATCCCTGAAGGGAACAACATATGGA | 336 | |
|  |  |  | *rDIII-WNV-R* | CTTGTCGACGCTGCTCCCAGATTTGTGCCA |  |  |
|  |  | | | | |  |

Restriction sites are depicted with underlined nucleotides. GATATC – *Eco*RV*,* GTCGAC – *Sal*I and GGATCC – *BamH*I

**Supplementary methods**

**Supplementary method S1**

## **Human brain microvascular endothelial cell (HBMECs) culture.**

Human brain microvascular endothelial cells were procured (ScienCell, USA). Cells were cultured in 25 cm^2^ cell culture flask coated with collagen type I (Sigma, USA) in complete EBM-2 medium (Lonza, UK) containing 10% FBS, 1.4 μM hydrocortisone (Sigma), 5 μg/mL ascorbic acid, 10 mM HEPES and 1 ng/mL bFGF (Sigma). Cells were incubated at 37°C in a humid atmosphere of 5% CO_2_ until confluence. Cells were passaged on 6 well cell culture plate covered with collagen type I (Sigma, USA) in complete EBM-2 medium. Cells from the monolayer (4^th^ passage) were used in the experiment. Cells were either harvested for RNA isolation (non-induced cell control) or incubated with rDIII of TBEV or WNV.

**Supplementary method S2**

**Selection and culture of clones for recombinant protein production.** The gene fragments encoding DIII of TBEV and WNV were amplified by PCR from cDNA. Information on primer, amplicon length and restriction enzymes used are presented in **Supplementary Table S2**. Amplified fragment was digested with restriction enzymes (*Eco*RV or *BamH*I and *Sal*I, Thermo Fisher Scientific, Slovakia), as per the manufacturer’s instructions. Digested fragments were ligated into pQE-30-mCherry-STOP plasmid. Please note that in this vector mCherry serves as stuffer sequence, which is cut out during the digestion of vector with restriction enzymes. Ligation mix contained 2µl of ligation buffer, 2µl of PEG 8000, 0.5 U of the T4 ligase, amplicon and plasmid (50 ng) in molar ratio of 10:1, and water up to 20 µl. Ligation was performed at 22°C for 1 hr. Ligation mix was purified using NucleoSpin (Macherey-Nagel, Germany) and transformed into *E. coli* M15 strain (Qiagen, Germany). Transformants were selected from LB agar plates (Lysogeny broth, Sigma; supplemented with 2% bacteriological agar, 1% glucose, 25 µg/mL kanamycin and 50 µg/mL carbenicillin). Presence of encoding gene in transformants was confirmed by sequencing.

A single colony carrying insert gene was cultivated in Terrific broth (TB, 15 g/L tryptone, 30 g/L yeast extract, 12.5 g/L NaCl, 2.5 g/L MgCl_2_/MgSO_4_, 100 µL/L metal mix, 7.5 mL/L glycerol) supplemented with 1% glucose, 50 µg/mL carbenicillin and 25 µg/mL kanamycin until OD_600_= 6. Bacterial cells were pelleted (centrifugation at 6,000 × g for 10 minutes) and resuspended in fresh TB medium without glucose. Protein expression was induced with 1 mM IPTG (Fermentas, Slovakia) at 30 °C for 8 hrs.

**Supplementary method S3**

**Purification of the rDIII.** After induction, cells were pelleted (17,880 × g for 10 minutes) and lysed in lysis buffer (0.03 M Na_2_HPO_4_, 0.5 M NaCl, 0.001% Tween 20, 10% glycerol, 1x protease inhibitor cocktail, Sigma-Aldrich) with four freeze-thaw cycles followed by sonication on ice (2 cycles; 30-s pulses, 100% amplitude). Proteins were purified with nickel affinity chromatography (Ni-NTA agarose beads, ABT agarose, Spain) as per manufacturer´s instructions. Briefly, Ni-NTA beads were equilibrated with the buffer (50 mM Na_2_HPO_4_, 300 mM NaCl, 0.001% Tween 20, pH 8). Beads were then incubated in the lysate for 1 hrs at 8°C with constant rotation (140 rpm). Unbound proteins were washed with washing buffer (50 mM Na_2_HPO_4_, 300 mM NaCl, 0.001% Tween 20, 20 mM imidazole, pH 8) for 5 times and protein were eluted in elution buffer (50 mM Na_2_HPO_4_, 300 mM NaCl, 250 mM imidazole, pH 8).

Proteins were immediately subjected to gel filtration (Sephadex G25, 30 ml column, *in-house* prepared) on ÄKTApurifier (GE-healthcare, 2 ml/min flow, max 0.45 MPa pressure) to remove imidazole and accomplish the buffer exchange. The buffer used for exchange was 50 mM Na_2_HPO_4_ (pH 7.0). NaCl was then added (to adjust the concentration between 0.1 - 0.2M) to the buffer-exchanged protein and loaded on modified-polylysine resin (High capacity endotoxin removal resin, Thermo Fisher Scientific) packed in 1 ml column (*in-house* column packing). Conditions for this chromatography performed on ÄKTApurifier were: both start and elution buffers (isocratic condition) – 25 mM phosphate buffer containing 0.1 M NaCl (pH 7.0, prepared in endotoxin free water), flow rate - 0.15 ml/min, pressure limit - 0.25 MPa maximum, sample load - 2 ml. Please note that eluate was collected in biosphere endotoxin free tubes (Sarsted, Germany) and protein was stored immediately in several aliquots until use.

**Supplementary method S4**

**SDS-PAGE and MALDI-TOF**

Whole cell lysate of *E. coli*, flow-through from nickel affinity chromatography and rDIII eluted after endotoxin removal were separated on SDS-PAGE. Briefly, the protein samples (5 µl each) were mixed with lithium dodecyl sulfate sample buffer (4x LDS sample buffer, Invitrogen, Slovakia) as per manufacturer’s instructions and incubated at 72°C for 15 min. Electrophoresis was carried out at 40 mA in 1x running buffer (20x NuPAGE MOPS SDS running buffer, Invitrogen) until the dye reached the bottom of the gel (10% Bis-Tris polyacrylamid 12 well gel, Invitrogen). Protein were stained with Coomassie as per manufacturer’s instructions (Bio-Rad).

For MALDI-TOF, 0.8 µl of the purified protein (after endotoxin removal) was mixed with 0.8 µl sDHB matrix (Bruker Daltonics, Germany; sDHB dissolved up to saturation in TA50 (50:50 [v/v] acetonitrile: 0.1% TFA in water). One microliter of the protein-matrix mix was spotted on the ground-steel plate (Bruker Daltonics) and allowed to air dry. Acquisition was performed in flexControl V 3.4 in linear mode with 60 Hz laser intensity (200 shots) on flex with reflectron MALDI mass spectrometer (Bruker Daltonics). Mass was analyzed in flexAnalysis V3.4 software of Bruker Daltonics by comparing it with calibrants (protein calibration kit I, Bruker Daltonics).

**Supplementary Method S5**

**Library preparation for RNA sequencing**

250 ng of RNA were reverse transcribed with oligodT primers for synthesis of the first strand cDNA using QuantSeq 3′ mRNA-Seq Library Prep Kit (Lexogen, Austria) as per manufacturer’s instructions. RNA template was removed and second strand was synthetized by using random hexamer containing Illumina-compatible linker sequences at its 5′ end. Double strand DNA library was purified using magnetic beads provided in the kit. Each library was amplified by PCR using unique single indexing i7 primers to add complete adapter sequence required for cluster generation and to generate sufficient DNA for sequencing and quality control. The number of cycles in PCR for each library was determined using PCR Add-on kit for Illumina (Lexogen). Number of cycles used for library amplification were as follows: HBMECs induced with rDIII-TBEV *-* 19 cycles, HBMECs induced with rDIII-WNV - 19 cycles and non-induced cells - 18 cycles. Amplified libraries were purified using magnetic beads supplied in the kit. Quality of the library and length of the fragments were checked on fragment analyzer. Libraries were sequenced on Illumina NextSeq, single-end 75 bp, to a minimal depth 8 million reads per sample.
